# Why did you choose psychiatry? a qualitative study of psychiatry trainees investigating the impact of psychiatry teaching at medical school on career choice

**DOI:** 10.1186/s12888-017-1445-5

**Published:** 2017-07-28

**Authors:** A. Appleton, S. Singh, N. Eady, M. Buszewicz

**Affiliations:** 1 Research Department of Primary Care and Population Health, University College London, London, NW3 2PF UK; 2 East London NHS Foundation Trust, 9 Alie Street, London, E1 8DE UK

**Keywords:** Education and training, Qualitative research, Stigma and discrimination, Careers in psychiatry

## Abstract

**Background:**

There is no consensus regarding the optimal content of the undergraduate psychiatry curriculum as well as factors contributing to young doctors choosing a career in psychiatry. Our aim was to explore factors which had influenced psychiatry trainees’ attitudes towards mental health and career choice.

**Method:**

Qualitative in-depth interviews with 21 purposively sampled London psychiatry trainees analysed using the Framework method.

**Results:**

Early exposure and sufficient time in undergraduate psychiatry placements were important in influencing psychiatry as a career choice and positive role models were often very influential. Integration of psychiatry with teaching about physical health was viewed positively, although concerns were raised about the potential dilution of psychiatry teaching. Foundation posts in psychiatry were very valuable in positively impacting career choice. Other suggestions included raising awareness at secondary school level, challenging negative attitudes amongst all medical educators, and promoting integration within medical specialties.

**Conclusions:**

Improvements in teaching psychiatry could improve medical attitudes and promote recruitment into psychiatry.

**Electronic supplementary material:**

The online version of this article (doi:10.1186/s12888-017-1445-5) contains supplementary material, which is available to authorized users.

## Background

With much recent media attention concerning the provision of good mental health services, there is a heightened awareness of the importance of having an engaged, competent workforce managing patients’ mental health problems with confidence. How medical students are taught psychiatry and their initial experiences of patients with mental illness are likely to be crucial factors determining attitudes towards this specialty and subsequent career choice [1, 2]. Despite this, there is still no consensus as to what constitutes the optimal undergraduate teaching experience in psychiatry and what other factors contribute to young doctors choosing a career in this specialty.

In the United Kingdom (UK) when doctors qualify from medical school they then proceed to complete two foundation years with different rotations in a range of medical specialties. In their second foundation year they apply for their choice of medical specialty, which will be a commitment to this next specialty for a number of years with a view to reach consultancy or the equivalent in this field. This is when they decide whether they want to specialize in psychiatry, general practice, core medicine or surgery among many other choices. This decision shapes their future career paths and there is an important period between commencing medical school and applying for these jobs where these medical students and doctors are receptive to influences in making their career choice. In psychiatry, there is limited knowledge of factors that help promote career choice into this specialty, in addition to what promotes positive attitudes to mental health in the future medical workforce in general; both of which are important as they affect the way healthcare will be delivered in future. To date there have been no qualitative studies looking at the views of psychiatry trainees on this topic.

There has been little or no change in the numbers of applicants to psychiatry in the UK over the years. In a national cohort study (1974–2009) 4–5% of doctors specified psychiatry as their first choice of future career at 1, 3 and 5 years after graduation [3]. Further work, including a review of undergraduate experiences identified an enthusiasm for psychiatry at secondary school that dissipated during medical school [3, 4]. Some of the reasons given included: insufficient exposure to psychiatry as an undergraduate, the quality of teaching received and the lack of status of psychiatry amongst peers [3]. Underlying stigma among medical students towards mental illness has been suggested as an influential factor in shaping the negative views that some students have towards a career in psychiatry [5–9], with the suggestion that it should be openly discussed with students in order to overcome this [9]. A recent British Journal of General Practice editorial [10] highlighted the need to “eliminate systematic denigration” of psychiatry at medical school, stating that parity of esteem for mental and physical health problems is the key to future recruitment – particularly at undergraduate level. The authors also highlighted the wider implications of this stigmatization on patient care and emphasized the importance of overcoming these negative attitudes towards mental illness within society in general [10].

Each UK medical school directs the content of their undergraduate psychiatry curriculum. Despite the Royal College of Psychiatrists providing a guide for the desired curriculum content [11] each UK medical school has control over the content of their own undergraduate psychiatry curriculum. In previous studies positive changes in their students’ attitudes towards psychiatry have been linked to seeing patients improve, being directly involved in their care and being actively encouraged by their consultants [2, 4]. The relationships between students and their teachers, positive role models and mentoring have been found to be as important as curriculum content and teaching methods in providing a positive experience of psychiatry [9, 12–14]. The optimum scheduling of psychiatry teaching within the undergraduate course is unclear. Some medical schools have moved towards integration of psychiatry teaching - that is the teaching of physical and mental components of illness occurring concurrently to reduce the separation in future doctors’ minds with regard to mental and physical health problems. Studies have reported pre-registration house officers taught with this approach as being more confident in managing psychological disorders [15, 16].

This study aimed to explore factors before, during and after medical school influencing attitudes towards mental health and a career choice in psychiatry. In-depth interviews were used to explore the views of psychiatry trainees about their undergraduate teaching experiences and to identify both barriers and positive factors influencing the selection of psychiatry as a career.

## Methods

### Design

In-depth qualitative interviews with a range of purposively sampled London based psychiatry trainees, aiming to explore their experiences of being taught psychiatry at medical school and how these may have affected their career choice.

### Participant selection and recruitment

Psychiatry trainees working within the London deanery were invited to complete an online survey in an email sent through the Royal College of Psychiatrists email database (results reported separately [17]). On completion of the survey trainees were asked to provide their details if they agreed to be considered for an in-depth interview. Those who agreed were sent an information sheet and further brief demographic questionnaire to assist in purposive sampling for the qualitative interviews, which was based on age, gender, ethnicity, stage of training and medical school attended.

### Conducting the interviews

Interested trainees who had been purposively selected were sent an information sheet and consent form prior to the in-depth interviews arranged at a convenient London site. All interviews were conducted by AA using a topic/interview guide (see Additional file 1 Topic guide) developed from reviewing the background literature and initial analysis of the survey results, and revised according to emerging themes from four pilot interviews conducted with a combination of junior doctors and psychiatry trainees. Reflection and ongoing discussions with a colleague (MB) took place between interviews to ensure standardization of interview style and minimize bias. Informed written consent was obtained prior to the interviews from all participants who were offered a £30 High Street Voucher in compensation for their time. Interviews were digitally recorded, anonymised and transcribed by AA.

### Data analysis

At least two members of the study team reviewed each transcript independently. AA coded all the data. The participants did not provide feedback on the transcripts due to concerns this could alter the findings. A thematic framework was developed and agreed by consensus after the research group had reviewed all the transcripts and identified key themes. Interview data was organised using Microsoft Excel charts and distributed amongst the research team (AA, MB, NE and SS) for familiarization and detection of themes, which were then finalised at a meeting attended by all members. The Framework approach was selected as it allowed the qualitative data to be organized in a transparent and systematic way [18]. Framework is a method for analyzing qualitative data, and allows both a case and theme based approach to the analysis, which helps reduce data by summarization [19] whilst still allowing researchers to compare data across and between cases [20]. It ensures links are retained to the original data which provides a comprehensive and transparent form of data analysis [19] useful when working in research teams. The results have been reported as per the criteria from the COREQ checklist for reporting qualitative research [21].

## Results

### In-depth interviews: sample characteristics

Twenty-one psychiatry trainees working in London who had qualified from 17 different UK medical schools were interviewed (see Table 1).

**Table 1:** Demographics of interview participants. Core training (CT) describes the first three years of psychiatry training when the trainees rotate between a range of subspecialties before choosing one to specialize in for their final three or four years of specialty training (ST). Only a few trainees complete an ST7 year and these trainees chose to subspecialize in two areas of psychiatry

Variable		n/21
Stage of training	Core Training	
	CT1	4
	CT2	3
	CT3	4
	Specialty Training	
	ST4	2
	ST5	4
	ST6	2
	ST7	2
Gender	Male	10
	Female	11
Ethnicity	White British	12
	Asian Indian	4
	Asian Pakistani	1
	Chinese	2
	Black African	1
	Other White	1
Age	20–30	8
	30–40	12
	40–50	1
Entry to medical school	Undergraduate	17
	Graduate	4

Interview length ranged from to 22 to 50 min and interviews continued until data saturation was reached which was defined in our study as when no new themes were emerging from the final five interviews.

The themes extracted from the interviews are summarized in Table 2 and described in detail below:

**Table 2:** Summary of themes and subthemes from the interviews

Summary of themes from the interviews	
Exposure to psychiatry at medical school	
• Length of placement	
• Early introduction of psychiatry	
• Temporal learning: Having time to see changes in patients	
• Flattened hierarchy: Feeling a valued member of the team	
• Receiving detailed feedback from supervisors	
Positive role models	
• Perceived personality traits among psychiatrists; for example, very approachable and people centered	
• Trainees were particularly influenced by consultants who a took personal interest in them and their development	
Views on integration of psychiatry teaching at medical school	
• Support for the concept of integration	
• Concerns about the dilution of psychiatry teaching overall	
Pathways in career decision-making: when did they decide on psychiatry?	
• Before medical school	
• During medical school	
• After medical school (Impact of foundation training)	
Appealing factors	
• Psychosocial factors	
• Practical factors; work life balance, skillset required, research potential	
Barriers	
• Negative attitudes/stigma	
• Isolation	
• ‘not a proper doctor’	
• Emotional stress and responsibility	
Recommendations	
• Buddy schemes	
• Mentoring	
• Raising awareness at secondary schools	
• Challenging negative attitudes at medical school	

### Important aspects of undergraduate experience affecting career choice

Many psychiatry trainees interviewed had taken some time after qualification to come to their decision to pursue a career in psychiatry, with both positive factors and potential barriers having an influential role.

A recurrent theme was the quality of their exposure to psychiatry at medical school*,* which was very important for some in influencing their attitudes and awareness of mental illness.

*P18 Male ST6: “I thought it was fantastic. I think out of all my clinical attachments, it was the one in which I'd felt most involved… my attitude…toward psychiatrists themselves became more positive. I had very little understanding of mental illness, prior to doing my psychiatry attachment”*

Some trainees identified how having a reasonable time on such a placement (i.e. 8 weeks) allowed them to see how patients might change or improve over time, which they viewed positively.

*P16 male ST5: “It has to be long enough so you can see changes in patients, so you don’t just see a snapshot”*

Several also suggested the benefit from having sufficient time to get involved in the team, which was considered important in creating a positive teaching environment and experience. A few trainees felt that as students they were valued members of the team during their psychiatry placements, which positively impacted on their attitudes and career choice and was not necessarily the case with teaching received in other specialties.

*P16 male ST5: “It was revolutionary to have someone who was interested in us as people”*

Some recommended earlier placements in psychiatry, in order to encourage students to consider psychiatry as a viable career path earlier in their time at medical school.

*P13 female CT1 “I like the idea of teaching it [psychiatry] earlier on in medical school and getting people comfortable with mental health and aware of psychiatry as an independent specialty in itself rather than a second choice.”*

However, some also described negative undergraduate experiences; particularly regarding the variability of their experience of psychiatry placements, with the experience commonly described as being ‘hit and miss’. Some trainees suggested that all students should have exposure to both inpatient and outpatient psychiatric experiences.

*P19 male ST5 “The clinical experience is variable… A lot of the places, they [medical students] get ignored or staff don’t have the time or inclination to teach…”*

### Positive role models

Role models and mentors had influenced many of the trainees in making their career choice.

*P20 female CT1: “The psychiatric consultants I interacted with [as a medical student] were role models that I wanted to follow; they were personable and approachable and very professional people who seemed to have time for everyone and I put a great value on that after having the experience in my foundation years where I felt quite intimidated by a lot of my seniors [in other specialties]”*

Some trainees suggested there might be particular personality traits, which were helpful in psychiatry:

*P18 male ST6: “I think that it attracts people who are more people-centered…genuinely interested in people's troubles and difficulties and…wider problems in society as well.”*

Several trainees recalled positive experiences of their undergraduate psychiatry teachers showing a particular interest in the students as individuals and doctors-to-be. Some trainees recalled how getting detailed personal feedback improved their confidence and skills.

*P17 female CT2: “I had a lot of encouragement in the sense of how I was doing and… advice about how to change things as well.”*

In addition some trainees had identified their own role models through undertaking special study modules in psychiatry or attending career talks with inspiring speakers who helped nurture their interest.

*P10 female CT2: “I’ve come into psychiatry purely because of extracurricular things that I organised at medical school…” “Attending a careers talk… out of the blue… with a charismatic speaker”...“I remember going home after this talk and saying to my family, that’s it, this is what I’m going to do.”*

### Integration of psychiatry within the medical curriculum

Most of the trainees had been taught in a block of psychiatry teaching, before curricula integrating teaching about mental health with physical health were introduced. Many of those interviewed favoured the idea of integration provided it did not detract from or dilute core psychiatry teaching to ensure its recognition as a specialty in its own right.

*P16 male ST5: “It is a delusion in a way or it is in denial not to teach those two things together”*

Some trainees suggested that the integration of psychiatry could be improved through increasing access to liaison psychiatry and primary care mental health placements. Some respondents suggested this would also help future psychiatrists to feel more confident with managing co-existent physical health problems and raise awareness of the overlap between physical and mental health disorders amongst all future doctors.

*P15 female CT3: “Psychiatry crosses all different aspects of people’s lives... it’s everybody, and the problem is that physical health of psychiatry patients is really poor, so it doesn’t make sense that they’re taught so separately when they have so many overlapping features”*

### Decision to choose a career in psychiatry

There were three main pathways that trainees had followed when deciding to enter into a career in psychiatry

1. Those who already had a prior interest and had decided to choose psychiatry before entering medical school.

P4 male CT1: *“I was drawn to psychiatry before going to medical school, so I suppose I was more open to [those] teaching placements”*

2) Those whose medical school experiences encouraged them to choose psychiatry, of whom, several had undertaken additional activities to explore their interest, such as special study modules, or specific career talks.

P18 male ST6*: “[My decision to choose psychiatry] … was partly because I had a very positive experience when I was a student, I thought that the job would be very interesting and rewarding and… that it would play to my strengths as well.”*

3) Those who had been interested in the possibility of psychiatry as a career, but did not firmly decide until after completing a Foundation year job in psychiatry.

P6 male CT1 *“I think if I hadn’t had those experiences during medical school… although I didn’t come away from this thinking, oh, I must definitely become a psychiatrist, it did make me think, well, I’d like to get more exposure to it, and it’s because of that, that I chose a Foundation programme with psychiatry. If I hadn’t had the Foundation programme experience, I probably wouldn’t have decided to pursue it”*

However, several respondents commented on how choosing psychiatry was often a decision made later in their career path, which has implications as Foundation doctors are expected to select their specialty of choice within a year of graduating from medical school.

*P1 female ST5: “I think I’m more likely to meet people who have done other things and then they’ve decided they want to do psychiatry, so it’s almost like a more mature decision in a way…”*

### Factors encouraging a choice of career in psychiatry

#### Psychosocial aspects

Many of the respondents found the opportunity to have more time with patients and involvement in the psychosocial aspects of their care appealing and said this had contributed to their decision.

*P5 female ST7 “It just felt like a nice human specialty that you were really listening to your patient and understanding what sort of problems they were going through. That’s what drew me to it really”*

#### Practical aspects

Several trainees described a healthy work-life balance as having influenced their choice of psychiatry:

*P15 female CT3 “There seems to be a larger emphasis on work-life balance in psychiatry, they encourage people to take time out …do things that interest them… it feels like they actually value their trainees”*

Feeling challenged to develop particular skills was a potential appeal for some:

*P5 female ST7 “There was something quite skillful about seeing these psychiatric patients and actually establishing a rapport with them and getting them to the right team…. that was the different kind of challenge”*

Respondents tended to rate either the psychosocial aspects or the scientific aspects of a career in psychiatry, but not both as factors that appealed to them. A few described the research potential of psychiatry as an attraction:

*P11 male ST7: “I think the fact that it's quite a young specialty… it's a field that's rapidly developing, lots of new research coming out… I felt like psychiatry feels like a bit of a new frontier…it's a dynamic area.”*

### Barriers to choosing a career in psychiatry

#### Negative attitudes

Most trainees described having had negative experiences during their medical student and Foundation training and all suggested that some stigma towards working with people with mental health difficulties remains prevalent. Many described having to overcome negative attitudes from family members, peers, and senior medical clinicians as barriers along their path to choosing a career in psychiatry

*P17 female CT2: “There is always a stigma. In my medicine F2 post ... I had just finished psychiatry and when I said that I wanted to do psychiatry my consultant said something like ‘then there is no point bothering with you then’.”…*

*“They [family] didn’t understand why I didn’t want a ‘nice GP job’ where people were ‘less dangerous’ and ‘less rude’ apparently. They wanted me to be the type of doctor who was highly regarded. And they felt psychiatrists were not highly regarded... But since me choosing it, people’s attitudes have changed slightly.”*

#### Isolation

The physical isolation from the rest of medicine was also considered a deterrent by some of the trainees.

*P6 male CT1: “Being on call in psychiatry as an F2, you …feel a bit isolated from other doctors”* “*Most of the psychiatry training happens in separate NHS trusts…”*

#### Not a ‘proper doctor’

There was a frequent concern expressed about losing medical skills and no longer being considered a ‘real doctor’ when choosing to specialize in psychiatry.

*P12 female ST6 “I think you get de-skilled quite quickly as a psychiatrist, because you don’t deal with physical health problems…You become more like a mental health specialist rather than a doctor... I think that discourages people from wanting to do it…they don’t get as much pride in being psychiatrists as…one would as a neurosurgeon.”*

#### Emotional stress and responsibility

Several trainees also identified emotional stress as a barrier and described how this could be precipitated by the responsibility of making decisions based on clinical assessments conducted by the psychiatric team together with their individual clinical judgment rather than also being able to rely on investigations, as in most other medical specialties.

*P14 female CT3: “It is about you and the decision you made with nothing to necessarily support you… If something goes wrong, it does feel it was a bit like you made that error of judgment. Yes, so maybe there's a degree of sort of responsibility there”*

### Recommendations for improving teaching in psychiatry and recruitment to the specialty

Buddy schemes, mentoring and shadowing opportunities for medical students and Foundation year doctors were all suggested as potentially helpful for those considering applying for psychiatry. Balint groups for medical students during psychiatry placements were also suggested.

There were several suggestions to improve the image of psychiatry in the media and with the public, as well as raising awareness at secondary school through outreach projects and work experience placements in psychiatry.

*P18 male ST6: “Psychiatry's just off the radar of a lot of teenagers who are going up to apply to university.”*

*P2 male ST4: “I don’t think psychiatry sells itself very well. We should be trying to I think demystify it bit, make it more human really.”*

Several respondents described the importance of challenging negative attitudes towards mental health amongst all clinical staff, particularly those involved in both undergraduate and postgraduate teaching.

*P18 male ST6 “I think one thing that has to happen is that the attitude of clinical staff [in all specialties] has to be more positive towards psychiatry. To avoid students possibly picking up a stigmatized approach to psychiatry, staff who are directly involved in medical education, should be targeted as a group who need to have their awareness raised… and should demonstrate a respect for mental health care.”*

## Discussion

### Main findings

This is the first study exploring the detailed views of psychiatry trainees regarding the factors, which helped or hindered their final choice of career pathway. Our interview results support the notion that psychiatry teaching at medical school does impact on career choice in psychiatry, although time may be needed after qualification for some to reflect on their final career choice. Psychiatry placements at medical school should be of a reasonable duration (at least 8 weeks) for students to see improvements in patients’ conditions and to feel a valued part of the team. Mental healthcare teaching should be integrated with physical health care and introduced early at medical schools to lessen the likelihood of negative attitudes developing and raise early awareness of psychiatry as a career option. Psychosocial aspects of the work and the opportunity to spend more time with patients were raised as positives by psychiatry trainees and could be promoted during undergraduate teaching. Barriers to choosing psychiatry as a career included negative attitudes that appear to still be deep seated within both the medical profession and general public. Positive role models were integral in promoting career choice for many in psychiatry and assisting in overcoming negative attitudes among peers/family/seniors. Foundation jobs in psychiatry had a clearly positive influence on career choice in psychiatry.

### Strengths and weaknesses of the study

We purposively sampled a representative group to obtain a range of views from psychiatry trainees, but are aware that we only sampled from the London psychiatry trainees who responded to a prior questionnaire survey; therefore our findings may not be generalizable nationally. We however ensured that the trainees interviewed had attended a wide range of medical schools in the UK. This study did not explore the perspectives of doctors who had decided not to pursue a career in psychiatry and were therefore a group who might have differing views about their medical school experiences. However, those interviewed did not all have a prior interest in psychiatry as undergraduates and had followed widely varying paths on the way to making their career choice. Those interviewed were a self-selected group so there may have been some element of respondent bias; however we purposively sampled and continued to data saturation defined, actively probing for negative or disconfirming views to overcome this where possible.

### Comparison with other studies in the field

Our findings, including recommending an 8 week placement of exposure to psychiatry at medical school has been supported by a recent Canadian study [22], which found that the duration of pre-clerkship exposure to psychiatry predicted the number of students selecting psychiatry as their first choice of discipline [22]. Respondents in our study also suggested that psychiatry should be introduced earlier in the medical school curriculum to mitigate against negative attitudes developing; this is supported by Brown et al. who concluded that earlier exposure promotes awareness and understanding of psychiatry amongst medical students [23]. Linking with the suggestion given by several of our participants the Royal College of Psychiatrists has expressed the aim to develop UK-wide medical student psychotherapy schemes by 2017 [24, 25] and to introduce Balint groups for all medical students, starting ideally at the time of their first contact with patients. This should help to develop empathic and therapeutic skills in all students, with evidence that it may also have a positive impact on recruitment to psychiatry [24, 25].

The expression of negative views at medical school by consultants in other specialties, which appears to particularly target psychiatry, is a form of stigmatization that needs to be overcome [10]. The trainees in our study described a general lack of awareness of psychiatry as a clinical specialty among school leavers and suggested promotion of outreach work into secondary schools to overcome this. A study reviewing psychiatry outreach programs to schools has identified their success; they appeared to have a positive influence on early career decision-making and raised awareness about the importance of seeking help for mental health issues [26]. Our interviews indicated how the psychosocial and scientific aspects of psychiatry appeared to appeal to different groups of trainees, supporting the notion of promoting both the social aspects of psychiatry and its research potential in order to attract more clinicians into psychiatry [27]. Finally, as foundation posts have been identified as having a positive impact on career choice this then supports a Royal College of Psychiatrists strategy for expansion of these posts throughout the UK. When fully implemented this would mean that almost half of all doctors will undertake a 4 month Foundation post in psychiatry - a significant increase [28] compared with now.

## Conclusion

### Meaning of the study and implications

Our study provides suggestions focused on improving the delivery of the recommended undergraduate psychiatry curriculum [11] including introducing psychiatry teaching earlier at medical school, for a duration of 8 weeks, and offering quality time in both inpatient and outpatient settings. Teaching about mental health problems should be considered throughout the curriculum, thus facilitating the teaching of psychiatry as an integrated subject combining the physical and psycho-emotional needs of patients. Negative attitudes within other medical specialties towards psychiatry need to be challenged and prevented from impacting adversely on students during medical school [10], in order to improve attitudes towards mental health amongst all future doctors and in wider society. Students should be offered buddies/mentors and taught by enthusiastic juniors as well as consultants; the critical importance of good role models cannot be overlooked in this context. Outreach work during secondary school should be extended to put psychiatry on the radar as a career choice. Foundation posts in psychiatry should be expanded to promote recruitment.

### Unanswered questions and future research and policy recommendations

Our results give clear recommendations for medical schools to incorporate into their psychiatry curricula, as well as those organising Foundation year training. It will be important to evaluate the impact of implementing the recommendations made with regard to the attitudes of all junior doctors towards mental health problems affecting their patients as well as recruitment to psychiatry. Further research is also required to examine the perspectives of junior doctors who decide against psychiatry as a career and those who choose not to pursue psychiatry beyond the initial stages of training. This will add to our understanding of what impacts on junior doctors choosing psychiatry as a career and hopefully assist in increasing recruitment figures as well as improving the way all doctors approach mental health problems in future.

## Additional file

Additional file 1: Topic guide - Topic/interview guide developed for conducting all interviews. (DOCX 19 kb)

## Abbreviations

CT: Core training
ST: Specialty training
UK: United Kingdom
