# Supplementary material for: Why did you choose psychiatry? a qualitative study of psychiatry trainees investigating the impact of psychiatry teaching at medical school on career choice
Source: BMC Psychiatry. 2017 Jul 28;17:276. doi: 10.1186/s12888-017-1445-5 (PMC5534074; doi:10.1186/s12888-017-1445-5)
Supplement: Additional file 1: — Topic guide - Topic/interview guide developed for conducting all interviews. (DOCX 19 kb) [file 12888_2017_1445_MOESM1_ESM.docx]

**Additional file: Topic guide**

1. **Introduction**

-Introductions by interviewer; background, workplace

-Explain:

-Nature and purpose of research

-Who research is for and funded by

-Introduce audio recorder

-Stress confidentiality and anonymity and the option of withdrawing at any time

-Confirm information sheet read and consent form signed

-Check whether any questions

1. **Detail on their undergraduate teaching in psychiatry**

- Where did they go to Medical School?

- What psychiatry teaching methods were used: lectures, ward/clinical, case discussions, role-play

-How much teaching on psychiatry did you have? (Block duration)

- When was it placed? Which year? Was it in a block? If so, how long was the block? Or was it integrated?

-Who taught psychiatry? And what were the teachers like; experience of good and bad teachers- explore

-Mentors – did anyone made a particular impression? Who? Why?

-Other activities that led to interest in psychiatry; elective, special study modules, groups- opportunities

-Attitude changes

1. **Views on their undergraduate teaching on psychiatry**

-What are your views (positive/negative) on the teaching of psychiatry?

-Attitudes to psychiatry before and after teaching – how did they change?

-Did the teaching of psychiatry influence your career choice?

-Was it stimulating/interesting/distressing/frightening?

-Explore experiences

1. **Understanding of the integrated curriculum**

-Understanding of this term

-Views on it and whether it is a good/bad idea and why

1. **Factors that led to them choosing psychiatry as a career**

–Graduate entry

–When did they decide: Before/during/after medical school

-Interest - Explore further- When did this begin what triggered it? What aspect interested them and why?

-Teaching factors

-Experience

-Role model/mentor

-Other factors that have contributed

-Personal factors- lifestyle, personal view or experience of mental health

-Colleagues/friends

-Workload perceptions

1. **Motivators and barriers to choosing a career in psychiatry (more generally)**

-Motivators to recruitment?

-Barriers to recruitment?

-Motivators for staying in psychiatry?

1. **Suggestions about improving recruitment from the participant**

-For the curriculum and teaching

-In general: any other ideas

1. **Views on being a psychiatry trainee**

-Are they happy with their decision?

-What have been their experiences since been a speciality trainee?

-Have they met their expectations?

-Have they met any trainees who have left psychiatry training? And if so, do they know why they left?

1. **Close the interview**
   1. Anything else to add?
   2. Thank the participant
   3. Any questions about the research?
   4. Reassure confidentiality and anonymity
   5. Check if they want to be informed of findings
